# Enhanced Immunogenicity of HIV-1 Envelope gp140 Proteins Fused to APRIL

**DOI:** 10.1371/journal.pone.0107683

**Published:** 2014-09-23

**Authors:** Gözde Isik, Kwinten Sliepen, Thijs van Montfort, Rogier W. Sanders

**Affiliations:** 1 Laboratory of Experimental Virology, Department of Medical Microbiology Center for Infection and Immunity Amsterdam (CINIMA), Academic Medical Center, University of Amsterdam, Amsterdam, the Netherlands; 2 Department of Microbiology and Immunology, Weill Medical College of Cornell University, New York, New York, United States of America; University of Missouri, United States of America

## Abstract

Current HIV-1 vaccines based on the HIV-1 envelope glycoprotein spike (Env), the only relevant target for broadly neutralizing antibodies, are unable to induce protective immunity. Env immunogenicity can be enhanced by fusion to costimulatory molecules involved in B cell activation, such as APRIL and CD40L. Here, we found that Env-APRIL signaled through the two receptors, BCMA and TACI. In rabbits, Env-APRIL induced significantly higher antibody responses against Env compared to unconjugated Env, while the antibody responses against the APRIL component were negligible. To extend this finding, we tested Env-APRIL in mice and found minimal antibody responses against APRIL. Furthermore, Env-CD40L did not induce significant anti-CD40L responses. Thus, in contrast to the 4-helix cytokines IL-21 and GM-CSF, the TNF-superfamily members CD40L and APRIL induced negligible autoantibodies. This study confirms and extends previous work and shows that fusion of Env-based immunogens to APRIL can improve Env immunogenicity and might help in designing HIV vaccines that induce protective humoral immunity.

## Introduction

A protective HIV-1 vaccine remains elusive. One of the priorities of HIV-1 vaccine research is the induction of broadly neutralizing antibodies (bNAbs) by recombinant HIV-1 envelope glycoproteins (Env). Induction of such antibodies (Abs) is challenging and so far no vaccine has been able to induce protective bNAbs against HIV-1. Env, which is the only target for bNAbs, has many properties to evade bNAb responses, such as (i) diversity, (ii) shielding of conserved bNAb targets by variable loops and conformational masking, (iii) protection by extensive glycosylation, and (iv) exposure of decoy epitopes on non-functional Env forms (reviewed in [1]). Moreover, the half-life of Abs raised with Env vaccination is unusually short (30–60 days) [2], [3]. As a result, the neutralizing Ab (NAb) response against Env vaccines is weak, narrow and short-lived.

As with any immunogen, the immunogenicity of Env vaccines can be increased by addition of costimulatory molecules (adjuvants), which can include molecules from the immune system. The wealth of well-defined costimulatory molecules in the immune system provides the opportunity to select ones that activate and skew the immune response towards the desired direction (i.e., humoral *vs.* cellular, mucosal *vs.* systemic, Th1 *vs.* Th2 or Th17, etc.) [4], [5]. We and others have been exploring vaccine strategies in which HIV-1 Env is directly fused to a costimulatory molecule. The direct fusion assures that the antigen and the adjuvant will not be physically separated and that both molecules will interact with the same immune cells. Such proteins include gp120 or gp140 fused to IFN-γ, TNF-α, Flt-3 ligand, CTLA4, C3d, β-defensin 2, CCL7, CCL22, IL-21, GM-CSF, APRIL, BAFF and CD40L [6]–[19].

One disadvantage of using self-molecules as adjuvants is the potential of inducing autoantibodies (autoAbs) that target or neutralize the native (self) cytokines. Although cytokines are included in many experimental vaccines and their beneficial effects are studied in detail, the elicitation of autoAbs is often not investigated. We have previously reported that high levels of anti-cytokine Abs were induced in mice and rabbits immunized with GM-CSF and IL-21 fused to HIV-1 Env gp140 [20]. Although such auto-responses might not necessarily be harmful, autoAb induction should be investigated when cytokines are included in vaccines.

We have previously shown that fusion of APRIL to the C-terminus of Env (Env_APRIL_) enhances the titers of binding and neutralizing Abs against Env in rabbits [13]. Furthermore, when allowed to bind to human naïve B cells, Env_APRIL_ induced the expression of activation-induced cytidine deaminase (AID) which is involved in somatic hypermutation (SHM), class switch recombination (CSR) and the gene conversion processes of immunoglobulin (Ig) genes [13], . The induction of AID by APRIL and the involvement of AID in SHM are highly relevant for HIV vaccine research because HIV-1 bNAbs are usually highly somatically mutated [24], [25]. Furthermore, APRIL stimulates B and T cell proliferation [26] and promotes long-term survival of plasma cells (PCs) [27], [28], which could contribute to the longevity of humoral responses against HIV-1. Finally, APRIL promotes Ig CSR from Cμ to Cγ and/or Cα giving rise to IgG- and IgA-secreting cells, respectively [29], and it has been suggested that APRIL is the major promoter of IgA production under physiologic conditions of antigen exposure [30], thereby contributing to mucosal Abs. In summary, APRIL has the potential to (i) increase Ab breadth and potency by supporting SHM, (ii) enhance Ab longevity by supporting long-lived PCs, and (iii) enhance mucosal immunity by supporting class-switching to IgA. All these properties are highly desirable for an HIV-1 vaccine.

APRIL, naturally expressed by neutrophils, monocytes, macrophages and dendritic cells (DCs), and by activated T and B cells [31], interacts with two different receptors: B cell maturation antigen (BCMA), which is exclusively expressed on B cells [32], [33]; and transmembrane activator and calcium modulator and cyclophilin ligand interactor (TACI), expressed on B cells and activated T cells [34]. BCMA and TACI are also receptors for B-cell activating factor (BAFF). APRIL also binds to heparin sulfate proteoglycans that facilitate APRIL cross-linking and activity [35]. Because of the complexity of the binding patterns of APRIL, the role of each individual signaling pathway in humoral immunity has been difficult to dissect, but there is evidence that TACI signaling promotes CSR [36], [37], while BCMA signaling contributes to the survival of long-lived PCs [28].

In this study, we wanted to confirm and extend our earlier observations that Env_APRIL_ improved anti-Env responses, which were made in only 4 rabbits and lacked statistical power [13]. Here we show that Env_APRIL_ was able to bind and signal through both BCMA and TACI receptors *in vitro*. Our new data with 12 immunized rabbits per group confirm that Env_APRIL_ indeed significantly augments the response against Env. Furthermore, we show that the APRIL domain of Env_APRIL_ induces negligible anti-APRIL Abs. Similar results were observed with Env_CD40L_ and these results were confirmed in mice. This study shows that fusion of Env immunogens to APRIL might be useful for the design of Env immunogens aimed at inducing protective humoral immunity.

## Materials and Methods

### Constructs

All constructs in this study were based on the codon-optimized stabilized HIV-1 JR-FL (subtype B) SOSIP.R6-IZ-H8 gp140 sequence (Env_wt_) that has been described in detail elsewhere [38]–[41]. Despite the presence of the SOSIP mutations, these Env trimers are not native-like as we recently published for BG505 SOSIP.664 gp140 [42]–[44], which was not available when this study was initiated. The constructs used here were based on the earlier generation JR-FL SOSIP.R6 and the addition of the GCN4-based trimerization domain (IZ) and APRIL or CD40L domains at the C-terminus renders them uncleavable [12], [40]. Uncleaved and cleaved gp140 trimers are antigenically and structurally different [45], [46]. Amino acid numbering is based on HXB2 gp160 according to convention. The generation of SOSIP.R6-IZ-APRIL fusion protein (termed Env-APRIL or Env_APRIL_) is described in detail previously [12], [13]. Env_APRIL_ had an expected molecular weight of around 157 kD (∼17 kD per APRIL monomer and ∼140 kD for Env gp140). Mouse homologues were cloned as described previously [18]. The following nomenclature was used for these Env fusion constructs: Env-human APRIL: Env_hAPRIL_; Env-rabbit APRIL: Env_rAPRIL_; Env-mouse APRIL: Env_mAPRIL_; Env-rabbit CD40L: Env_rCD40L_; Env-mouse CD40L: Env_mCD40L_. Soluble recombinant cytokines encoding the sequences of human APRIL (hAPRIL), rabbit APRIL (rAPRIL), mouse APRIL (mAPRIL), rabbit CD40L (rCD40L), and mouse CD40L (mCD40L) were flanked with a His-tag (8 x histidine) that was cloned downstream the tPA leader at the N-terminus of the cytokine. The sequences of all constructs were verified prior to use.

### Reagents

Monoclonal Abs (MAbs) were obtained as gifts, or purchased, from the following sources: PA1, Dr. Bill Olson, (Progenics Pharmaceuticals, Tarrytown, NY); VRC01, Drs. Peter Kwong and John Mascola (Vaccine Research Center, Bethesda, MD); PGT121, PGT126 and PGT135, Dennis Burton (The Scripps Research Institute, La Jolla, CA); human BCMA-Fc and human TACI-Fc, R&D Systems (Abingdon, UK); MAb Aprily-5 to APRIL (mouse-anti-human, ALX-804-801), Enzo Life Sciences (NY, USA) and anti-His MAb (mouse, cat no: A00612), Genscript (NJ, USA).

### Cells lines and transfections

293T cells were maintained in Dulbecco's Modified Eagle's Medium (DMEM; Invitrogen, Breda, the Netherlands) supplemented with 10% heat inactivated fetal calf serum (FCS; HyClone, Perbio, Etten-Leur, the Netherlands), MEM nonessential amino acids (0.1 mM; Invitrogen, Breda, the Netherlands) and penicillin/streptomycin (both at 100 U/ml). 293T cells were transiently transfected with plasmids expressing recombinant Env using the Lipofectamine reagent following the manufacturer's recommendation (PAA, Pasching, Austria). Env containing supernatants were harvested 48 h after transfection and frozen in aliquots. Jurkat-BCMA-Fas-2309 cl13 (BCMA:Fas) [47] and Jurkat-JOM2 TACI-Fas 2455 cl112 (TACI:Fas) cells [48] were kind gifts from Pascal Schneider (University of Lausanne, Switzerland) and were cultured in RPMI 1640 (Invitrogen) with 10% FCS.

### SDS-PAGE, blue native PAGE and western blotting

SDS-polyacrylamide gel electrophoresis (SDS-PAGE) and western blotting were performed as previously described [40]. Env was detected using MAb PA1 (0.2 µg/ml) and HRP-labeled goat anti-mouse IgG (1∶5000; Kirkegaard & Perry Laboratories, Maryland, USA) followed by the Western Lightning ECL system (PerkinElmer, Groningen, the Netherlands) and His-tagged cytokines (hAPRIL, rAPRIL and rCD40L) were detected using anti-His MAb (0.25 µg/ml) followed by HRP-labeled goat anti-mouse IgG (1∶5000; Kirkegaard & Perry Laboratories).

### Immunoprecipitation assays

Immunoprecipitation assays were performed as previously described [13], using VRC01, PGT121, PGT126 or PGT135 at final concentrations of 3 µg/ml. The immunoprecipitates were analyzed by SDS-PAGE followed by western blotting with MAb PA1.

### Anti-gp120 ELISA

Anti-gp120 Ab titers were measured by D7324-capture ELISA as described previously [40]. In brief, Microlon 96-well plates (Greiner Bio-One, Alphen aan den Rijn, the Netherlands) were coated with anti-gp120 Ab D7324 (10 µg/ml) in 0.1 M NaHCO_3_ (pH 8.6; 10 µg/ml), 100 µl/well and incubated overnight (o/n) at 4°C. The plates were washed twice with Tris-buffered saline (TBS), and incubated with 2% skim milk powder (Sigma-Aldrich, Zwijndrecht, the Netherlands) in TBS for 30 min to block the residual protein-binding sites. After a TBS wash step, JR-FL gp120 protein from transiently transfected 293T cells was added to the wells for 2 h at room temperature and the plates were washed twice with TBS. Heat inactivated (30 min, 56°C) rabbit or mouse sera were serially diluted in TBS containing 20% sheep serum (Biotrading, Mijdrecht, Netherlands) and 2% skim milk powder, and incubated for 2 h. All serum samples were tested in duplicate. HRP-labeled goat anti-rabbit IgG or HRP-labeled goat anti-mouse IgG (Jackson Immunoresearch, Suffolk, UK) was used as secondary Ab and added for 60 min at a 1∶3000 dilution (final concentration 0.33 µg/ml) in 2% skim milk powder in TBS, followed by five washes with TBS/0.05% Tween-20 (TBS-T). Colorimetric detection was performed using a solution containing 1% 3, 3′, 5, 5′-tetramethylbenzidine (Sigma-Aldrich), 0.01% H_2_O_2_, 100 mM sodium acetate and 100 mM citric acid, and stopped by adding 0.8 M H_2_SO_4_. Absorption was measured at 450 nm. Midpoint titers (the serum dilutions at which the optical density was 50% of the maximum value) were calculated for all serum binding studies. When serum titration did not yield reliable sigmoidal curves with an upper plateau (i.e. in the case of anti-APRIL responses), endpoint titers (the serum dilutions at which the optical density (OD_450_) was three times above the background OD_450_ signal (without animal sera)) were also calculated. Both midpoint and endpoint titers were calculated using GraphPad Prism (version 5.03).

### Total IgG/IgA/IgM ELISA

Animal sera, treated as described above for the anti-gp120 ELISA, were diluted and coated o/n onto Microlon 96-well plates (half-area, Microlon 600, Greiner Bio-One) in 0.1 M NaHCO_3_ (50 µl/well). The plates were washed twice with TBS, and incubated with 2% skim milk powder in TBS for 30 min to block the residual protein-binding sites. The wells were incubated with HRP-labeled goat anti-rabbit IgG (1∶3000; Jackson ImmunoResearch), goat anti-rabbit IgA (1∶10000; Bethyl Laboratories, Montgomery, USA) or goat anti-rabbit IgM (1∶4000; Southern Biotech) in 2% skim milk powder in TBS and the subsequent steps were performed as described for the anti-gp120 ELISA.

### Anti-trimer ELISA

The anti-trimer ELISA was performed as previously described [18], [40]. Briefly, supernatants containing His-tagged Env_wt_ were diluted 1∶3 in TBS supplemented with 10% FCS and added for 2 h to pre-blocked Ni-NTA HisSorb 96-well plates (Qiagen, Venlo, the Netherlands). After three washes with TSM (20 mM Tris, 150 mM NaCl, 1 mM CaCl_2_, 2 mM MgCl_2_), serially diluted animal sera treated and diluted as described above for the anti-gp120 ELISA, were then added for 2 h followed by three washes with TSM 0.05% Tween-20 (TSM-T). HRP-labeled goat anti-rabbit IgG or goat anti-mouse IgG (all from Jackson ImmunoResearch) was added for 1 h at a 1∶3000 dilution (final concentration 0.33 µg/ml) in TSM plus 5% BSA, followed by 5 washes with TSM-T. The subsequent steps of the assay were identical to those of the anti-gp120 ELISA.

### Anti-APRIL and anti-CD40L ELISA

The anti-APRIL and anti-CD40L ELISA were performed as described previously for anti-IL-21 and anti-GM-CSF [20] and as described above for the anti-trimer ELISA [40]. The wells were coated with supernatants containing APRIL or CD40L (murine or rabbit), supernatant from untransfected cells (mock media), or Env_wt_ (for comparative purposes) and the plates were washed twice with TBS. Animal sera treated and diluted as described above for the anti-gp120 ELISA were incubated for 2 h and the subsequent steps were as described for the anti-gp120 ELISA [40]. The human APRIL control ELISA was performed using hAPRIL and serially diluted Aprily-5 to human APRIL (final concentration 1.0 µg/ml, ALX-804-801) (Enzo Life Sciences, NY, USA) as the primary Ab.

### BCMA and TACI binding ELISA

Binding of Env-APRIL proteins to the BCMA or TACI receptors was tested using ELISA as described previously [20]. Human BCMA-Fc or human TACI-Fc (with the Fc domain from human IgG1) were coated o/n on Microlon 96-well plates (Greiner Bio-One) at concentrations of 1.0 µg/ml and 2.0 µg/ml, respectively, in 0.5 M sodium bicarbonate buffer (pH 9.5) at 4°C. Plates were blocked with 5% BSA in TBS for 1 h at 37°C and washed three times with TBS. 3-fold serially diluted supernatants containing Env_APRIL_ proteins or control proteins were added and after 2 h incubation at 37°C the plates were washed three times with TBS-T and incubated with PA1 Ab (1.0 µg/ml in TBS) for 2 h at 37°C. Bound Env_APRIL_ was detected with HRP-labelled goat anti-mouse IgG (Jackson Immunoresearch) used at 1∶5000 (0.2 µg/ml). The subsequent steps were identical to the anti-gp120 ELISA.

### BCMA and TACI signaling assay

BCMA:Fas and TACI:Fas Jurkat reporter cells have chimeric BCMA and TACI receptors, respectively, coupled to the transmembrane and intracellular domains of Fas. Binding of APRIL to the receptors triggers the proapoptotic Fas signaling pathway that leads to cell death [47]. For killing assays, BCMA:Fas or TACI:Fas cells (3×10^4^ in 50 µl/well) were seeded in a 96-well flat-bottom plate and subsequently stimulated with 50 µl of serially diluted (3-fold) supernatants containing Env_wt_, Env_hAPRIL_, Env_rAPRIL_ or APRIL proteins for 16 h at 37°C. For the TACI signaling assays, Env_wt_, Env_hAPRIL_, Env_rAPRIL_ or APRIL containing supernatants were concentrated 8-fold (to a volume of 50 µl) prior to the experiment using centrifugal filters with a 30 kD molecular weight cutoff following the manufacturer instructions (Amicon, Merck Millipore, Amsterdam, the Netherlands). 20 µl of a solution containing 3-(4,5-dimethylthiazol-2-yl)-5-(3-carboxymethoxyphenyl)-2-(4-sulfophenyl)-2H-tetrazolium (MTS) and phenazine methosulfate (PMS) (1∶20, from CellTiter 96 Aqueous Non-Radioactive Cell Proliferation Assay, Promega, Madison, USA) was then added and the plates were incubated for 4 h. MTS is bioreduced by metabolically active cells into formazan which was detected by measuring the absorbance at 490 nm using an ELISA plate reader. The absorbance at 490 nm is directly proportional to the number of living cells in the culture.

### Neutralization assays

The TZM-bl reporter cell line was obtained through the NIH AIDS Research and Reference Reagent Program, Division of AIDS, NIAID, National Institutes of Health (John C. Kappes, Xiaoyun Wu, and Tranzyme Inc. [Durham, NC]) [49], [50]. All sera were heat inactivated (30 min, 56°C) before use. Neutralization assays were performed using the tier 1A viruses SF162 and MN/H9 and the tier 2 viruses WITO, TRO.11 and JR-FL. The latter virus is homologous to the vaccine antigen. The tier categorization of viruses according to neutralization sensitivity was based on ref [51]. Single cycle infection and inhibition experiments were performed as described previously, using 3-fold serially diluted sera, with each sample tested in duplicate against each virus [18], [20]. The percentage of neutralization was determined by measuring the reduction of the luciferase signal by each serum dilution compared to the values measured in the absence of serum (defined as 100%). The 50% (midpoint) neutralization titers were determined using GraphPad Prism (version 5.03).

### Immunizations

Plasmids encoding Env proteins were amplified using *DH5α* cells and isolated using an EndoFree Plasmid Giga kit (Qiagen, Venlo, the Netherlands). Two identical but independent immunization experiments were performed in New Zealand White rabbits. In each experiment 6 rabbits were immunized with Env_wt_ and 6 with Env_rAPRIL_. The experiments were analyzed simultaneously yielding 12 animals/group for each immunogen. Rabbits were immunized in the abdominal dermis at weeks 0, 2, 4 and 8 with 125 µg of endotoxin-free DNA using gene gun technology [52]. Blood samples were obtained at weeks 0, 2, 4, 6, 8 and 10, with a final bleed at week 12. Immunizations were carried out under contract by Genovac (Freiburg, Germany), at the facilities of Davids Biotechnologie (Regensburg, Germany). All animals were kept according to the regulations of the German Welfare Act of 19 May 2006 (BGBI I S. 1206), the regulations of the European Union guidelines (86/609/EWG of 24 November 2006), and the European Agreement of 18 March 1986 for the protection of animal trials and other scientific studies using vertebrates (Act of 11 December 1990 [BGBI II S.1486]). All protocols dealing with animal manipulations were in accordance with guidelines published by FELASA (Federation of European Animal Science Association) and GV-SOLAS (German Society of Laboratory Animal Science) and were reviewed and approved by the Davids Biotechnologie animal care committee. Sera from rabbits immunized with Env_rCD40L_ and the control Env immunogen were from a previous study [13], as were sera from mice immunized with Env_mAPRIL_, Env_mCD40L_ and their control Env [18].

## Results

### Env_APRIL_ binds to and signals through BCMA and TACI

**Figure 1:**
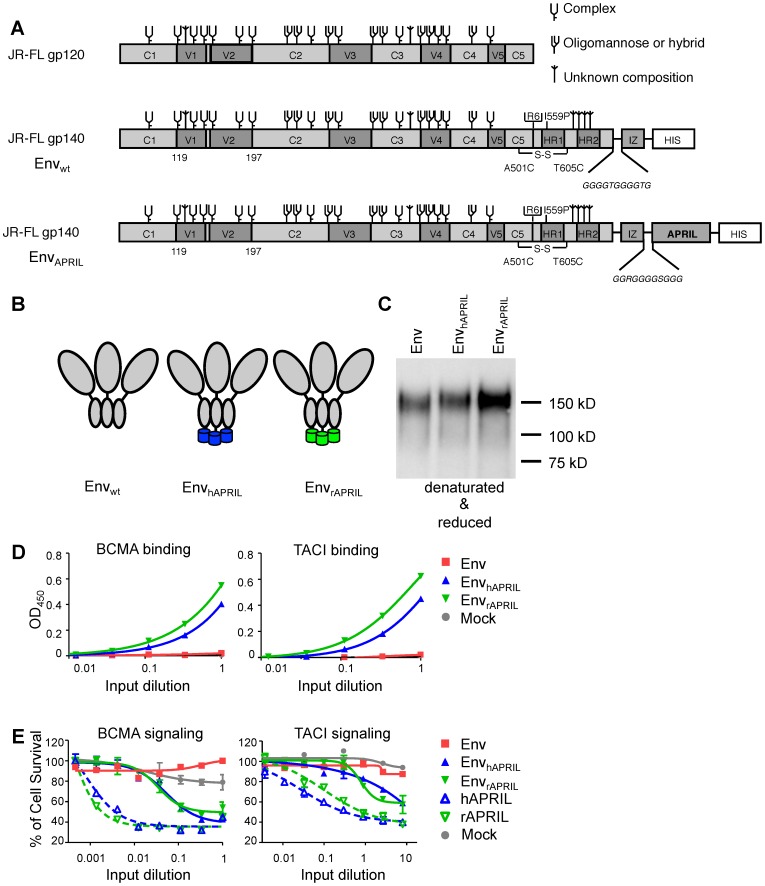
Schematics, expression and activity of Env_APRIL_. (A) Linear and (B) cartoon representation of Env_wt_, Env_hAPRIL_ and Env_rAPRIL_. The colors for hAPRIL (blue) and rAPRIL (green) are also used in the following figures. (C) The Env_wt_, Env_hAPRIL_ and Env_rAPRIL_ proteins were expressed transiently in 293T cells and analyzed by reducing SDS-PAGE followed by western blotting using MAb PA1. The migration of marker proteins is indicated. Env_wt_ migrated at the expected apparent m.wt. of 140 kD, while Env_hAPRIL_ and Env_rAPRIL_ migrated slightly slower because of the addition of APRIL (∼17 kD). (D) Binding of Env_wt_, Env_hAPRIL_, Env_rAPRIL_ and mock supernatants to human BCMA-Fc and TACI-Fc by ELISA. (E) Signaling induced by Env_hAPRIL_, Env_rAPRIL_ and controls in BCMA:Fas and TACI:Fas reporter cells as measured by a reduction of cell survival. Each condition was tested in duplicate and the results shown are representative for three independent experiments using proteins derived from three independent transfections.

Human, rabbit or mouse APRIL (hAPRIL, rAPRIL, mAPRIL) were previously fused to the C-terminus of HIV-1 Env gp140 (Fig. 1A and B) [12], [13], [18]. We recently described a native-like soluble Env trimer, BG505 SOSIP.664 [42]–[44], [46]. That protein was not used here, because it was not yet available when this study was initiated. Instead, the gp140 used here was based on the uncleaved JR-FL SOSIP.R6-IZ gp140 [38]–[41]. The chimeric molecules, denoted Env_hAPRIL_, Env_rAPRIL_ and Env_mAPRIL_, respectively, are secreted from mammalian cells, and form trimers efficiently but they are uncleaved (Fig. 1B and C, and data not shown) [13], [18]. These proteins bind CD4 and NAbs against conformational epitopes that constitute protein and glycan (Fig. S1 and [13]) and activate B cells to secrete IgM, IgG and IgA, and induce AID, the enzyme responsible for SHM [13]. To expand on this *in vitro* work, we investigated whether the APRIL domain of Env_APRIL_ is able to interact and signal through its two main receptors: BCMA and TACI. We first performed an ELISA in which we coated the plates with BCMA-Fc and TACI-Fc [53] and tested the reactivity of supernatants from 293T cells expressing Env_wt_, Env_hAPRIL_, Env_rAPRIL_, and as a control, mock media. Both Env_hAPRIL_ and Env_rAPRIL_ interacted efficiently with the human BCMA and TACI whereas there was no binding of Env_wt_ or mock (Fig. 1D).

Next, we evaluated whether Env_APRIL_ had the capability to induce signaling through BCMA and TACI. For this we employed BCMA:Fas and TACI:Fas reporter cells, which contain the extracellular domain of BCMA and TACI, respectively, fused to the transmembrane and intracellular domains of Fas [47], [48]. Ligation of APRIL to BCMA:Fas or TACI:Fas triggers the proapoptotic Fas signaling pathway, resulting in cell death. These cells were cultured in the presence of Env_wt_, Env_hAPRIL_, Env_rAPRIL_, hAPRIL and rAPRIL containing supernatants or mock supernatant (See Methods). Env_hAPRIL_ and Env_rAPRIL_ molecules induced cell death of both BCMA:Fas and TACI:Fas cells, as did the hAPRIL and rAPRIL controls, whereas Env_wt_ did not have an apoptotic effect on these reporter cells (Fig. 1E). The increased activity of hAPRIL and rAPRIL supernatants compared to Env_hAPRIL_ and Env_rAPRIL_ supernatants might be explained by the increased APRIL expression levels in hAPRIL and rAPRIL supernatants. The induction of apoptosis was also assessed visually. Both cells formed clumps in the presence of mock or Env_wt_ media, whereas apoptotic blebs were formed in the presence of Env_hAPRIL_, Env_rAPRIL_, hAPRIL and rAPRIL (Fig. S2). Thus, the APRIL domain of chimeric Env_APRIL_ constructs is able to interact with and signal through the two APRIL receptors BCMA and TACI.

### Env_rAPRIL_ induces an enhanced Env-specific antibody response in rabbits

**Figure 2:**
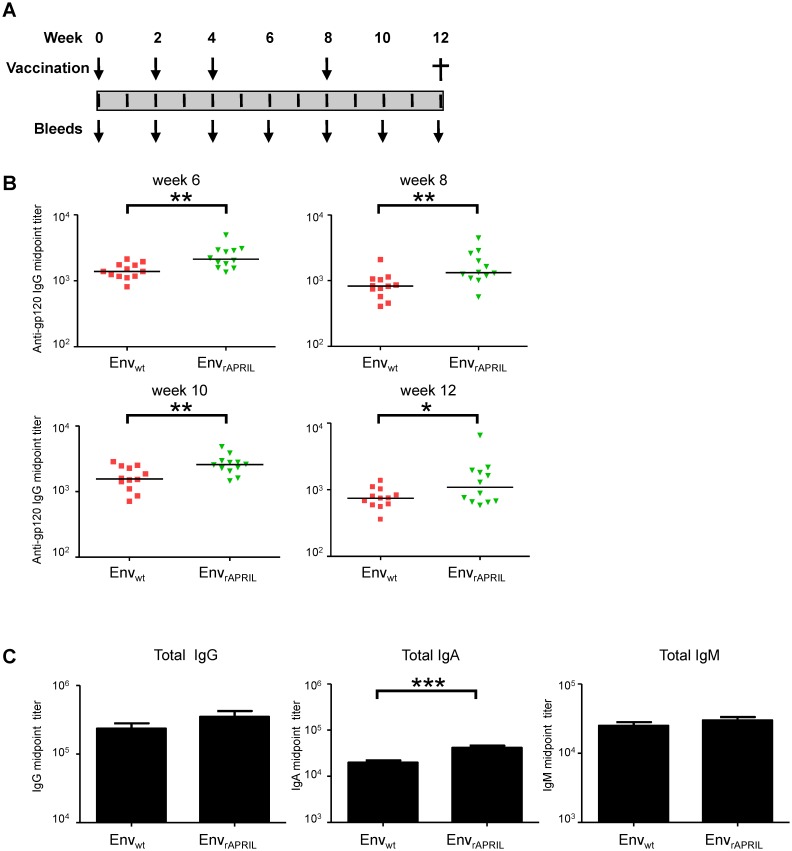
Env_rAPRIL_ induces an enhanced Env-specific antibody response in rabbits. (A) Immunization scheme. Two groups (n = 12) of rabbits were immunized via gene gun immunization, one group receiving Env_wt_ and the other Env_rAPRIL_. (B) Midpoint anti-gp120 IgG titers at week 6, 8, 10 and 12 as determined by ELISA. All sera were tested in duplicate with the mean values shown.*: p<0.05; **: p<0.01 (one-tailed Mann-Whitney test). (C) Total IgG, IgA and IgM midpoint titers in week 10 sera of rabbits immunized with Env_wt_ and Env_APRIL_.

We previously showed in 4 rabbits immunized with Env_rAPRIL_ that the fusion to APRIL enhanced the Env-specific immune responses. To extend these findings, rabbits were immunized with DNA encoding Env_wt_ (n = 12) and Env_rAPRIL_ (n = 12) via gene gun immunization at weeks 0, 2, 4, and 8, and the animals were bled at weeks 0, 2, 4, 6, 8, 10 and 12 for serological analysis (Fig. 2A). The anti-gp120 IgG levels were measured by ELISA. The anti-gp120 IgG midpoint titers of the Env_rAPRIL_-immunized rabbit sera were significantly higher than sera from animals vaccinated with Env_wt_ (Fig. 2B). The total IgG, IgA and IgM levels were increased in Env_APRIL_ immunized animals compared to Env_wt_ animals at week 10 and this was significant for IgA (Fig. 2C). The HIV-1 neutralization capacity was tested using pseudotyped viruses on TZM-bl reporter cells in a single-cycle assay [18], [20]. The neutralization-sensitive (tier 1) viruses SF162 and MN could both be neutralized by most of the rabbit sera (from week 10), but the more neutralization-resistant (tier 2) viruses JR-FL, WITO and TRO.11 could not be (Table 1) [51]. There were no significant differences in neutralization capacity between the Env_wt_ and Env_rAPRIL_ groups. Neutralization of tier 2 viruses would be very unexpected with such a short DNA only immunization protocol. Furthermore, the induction of tier 2 neutralization, even autologous tier 2 neutralization, awaits the availability of more sophisticated Env immunogens than the one used here. These immunization studies confirm that fusion of rAPRIL to Env improves the Env-specific Ab response in rabbits.

**Table 1:** 50% neutralization titers of sera from rabbits immunized with Env_wt_ and Env_rAPRIL_.

		Viruses				
Immunogen	Animal	SF162 (tier 1)	MN/H9 (tier 1)	JR-FL (tier 2)	WITO (tier 2)	TRO.11 (tier 2)
Env_wt_	543	29	113	<20	<20	<20
	544	141	817	<20	<20	<20
	545	211	789	<20	<20	<20
	546	177	4213	<20	<20	<20
	547	20	314	<20	<20	<20
	548	94	911	<20	<20	<20
	598	111	873	<20	<20	<20
	599	29	50	<20	<20	<20
	600	<20	99	<20	<20	<20
	601	<20	1919	<20	<20	<20
	602	274	842	<20	<20	<20
	603	170	609	<20	<20	<20
Env_rAPRIL_	555	87	179	<20	<20	<20
	556	25	2241	<20	<20	<20
	557	<20	41	<20	<20	<20
	558	76	2503	<20	<20	<20
	559	132	572	<20	<20	<20
	560	74	1468	<20	<20	<20
	616	<20	54	<20	<20	<20
	617	64	308	<20	<20	<20
	618	22	202	<20	<20	<20
	619	<20	49	<20	<20	<20
	620	23	281	<20	<20	<20
	621	92	439	<20	<20	<20

The categorization in neutralization sensitive viruses (tier 1 viruses) and neutralization resistant viruses (tier 2 viruses) is based on reference [51].

### Env_APRIL_ and Env_CD40L_ induce minimal anti-APRIL and anti-CD40L responses in mice and rabbits

**Figure 3:**
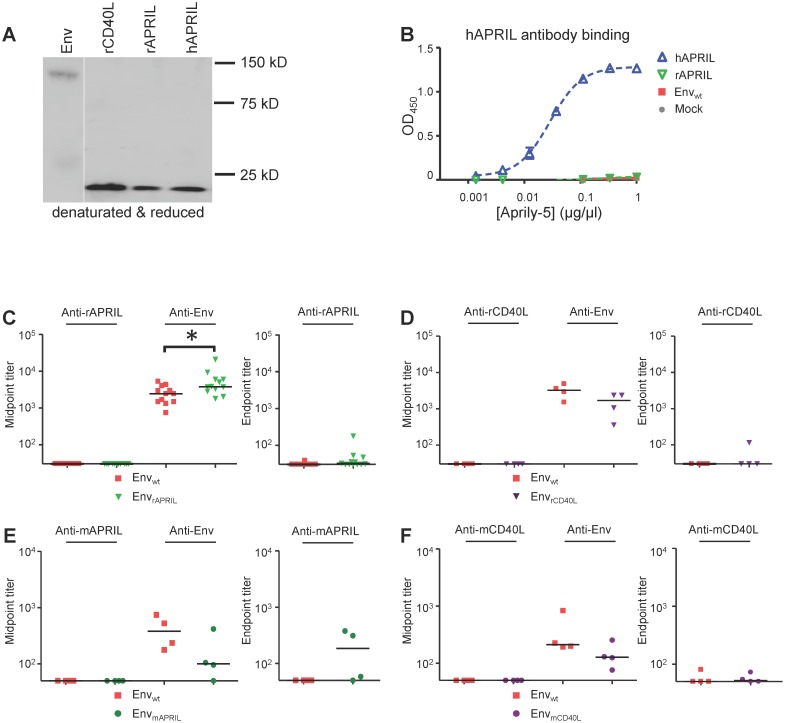
Env_APRIL_ and Env_CD40L_ induce minimal anti-APRIL and anti-CD40L responses in mice and rabbits. (A) Reducing SDS-PAGE analysis of Env_wt_, hAPRIL, rAPRIL and rCD40L proteins followed by western blot using an MAb against the His-tag. The migration of marker proteins is indicated. Env_wt_migrated at the expected apparent m.wt. of 140 kD, while the migration pattern of hAPRIL, rAPRIL and rCD40L was as expected based on their size of ∼17 kD. (B) Detection of hAPRIL by ELISA using Ab Aprily-5. (C) Midpoint titers of anti-rAPRIL and anti-gp140 Abs (left panel) and endpoint titers of anti-rAPRIL Abs (right panel) of week 10 sera from rabbits immunized with Env_wt_ or Env_rAPRIL_ (n = 12). (D) Midpoint titers of anti-rCD40L and anti-gp140 Abs (left panel) and endpoint titers of anti-rCD40L Abs (right panel) of week 12 sera from rabbits immunized with Env_wt_ or Env_rCD40L_ (n = 4). These 8 rabbits are described in reference [13]. (E, F) Midpoint titers of anti-mAPRIL (E), anti-mCD40L (F) and anti-gp140 Abs (left panels) and endpoint titers of anti-mAPRIL (E), anti-mCD40L Abs (F) (right panels) of week 8 sera from mice immunized with Env_wt_, Env_mAPRIL_, Env_mCD40L_ (n = 4). The mice were from the study described in reference [18]. All sera were tested in duplicate with the mean values shown.

We have previously reported on the strong induction of anti-cytokine responses with the chimeric proteins Env_IL-21_ and Env_GM-CSF_, which are similar in design to the Env_APRIL_ described here [20]. Therefore, we looked for the presence of anti-APRIL Abs in sera from rabbits immunized with Env_rAPRIL_ construct using a similar ELISA as we described previously for IL-21 and GM-CSF (see Materials and Methods and [20]). APRIL molecules were expressed efficiently and hAPRIL was recognized efficiently by the anti-hAPRIL Ab Aprily-5 in ELISA (Fig. 3A and B). Furthermore, both hAPRIL and rAPRIL were able to induce apoptosis of BCMA:Fas and TACI:Fas cells, indicating that they were well-folded and functional (Fig. 1D). Week 10 sera from animals immunized with DNA encoding Env_wt_ or Env_rAPRIL_ were tested in parallel for the presence of anti-rabbit APRIL Abs and anti-trimeric Env Abs. Env_rAPRIL_ induced higher titers of anti-Env Abs compared to Env_wt_ immunized rabbits (Fig. 3C), consistent with the gp120 ELISA results (Fig. 2B). Anti-rAPRIL responses were absent in Env_wt_ immunized rabbits, and minimal in the Env_rAPRIL_ rabbits (Fig. 3C). These results were in stark contrast with the high anti-rIL-21 and anti-rGM-CSF responses we previously observed in rabbits immunized with Env_GM-CSF_ and Env_IL-21_ [20].

To investigate whether the lack of anti-cytokine Abs was specific to APRIL, we tested historical sera from rabbits immunized with Env_rCD40L_ [13] for the presence of anti-rCD40L Abs using immobilized rCD40L (Fig. 3A). The Env_rCD40L_ immunized rabbits did not induce anti-rCD40L Abs (Fig. 3D). In order to exclude the possibility that the lack of anti-cytokine responses was species-specific, we also tested mice sera from a previous mouse immunization experiments in which mice were immunized with Env_mAPRIL_ and Env_mCD40L_ [18]. Env_mAPRIL_ and Env_mCD40L_ induced minimal anti-cytokine response in mice (Fig. 3E and F). We note that the anti-Env titers were lower in mice than in rabbits, which probably reflects differences in study regimen and duration. In conclusion, the TNF-superfamily members APRIL and CD40L fused to C-terminus of Env induce minimal anti-cytokine responses in mice and rabbits, in contrast to the 4-helix bundle cytokines IL-21 and GM-CSF.

## Discussion

HIV-1 Env, which is the only relevant HIV-1 antigen for the induction of bNAbs that can prevent HIV-1 infection, is a poor immunogen. The immunogenicity of poor immunogens such as HIV-1 Env can be enhanced by targeting specific immune cells. The majority of such targeting approaches have focused on DCs, which play an important role in induction of immune responses, especially T-cell responses [54]–[56]. DCs take up, degrade, and present the antigen to T cells thus contributing in particular to cellular immunity. However, the process of induction of bNAbs (also) requires interactions of naïve B cells with intact (i.e. unprocessed) antigen. The goal of this study was to target intact Env to B cells, whilst simultaneously providing a strong co-stimulatory signal to these B cells. Covalent linkage of Env to the TNF superfamily members such as APRIL and CD40L has been shown to be highly effective in activating human B cells and inducing the secretion of IgG, IgA and IgM *in vitro*, as well as increasing the anti-Env responses in rabbits [12], [13].

Here, we first assessed whether Env_APRIL_ could bind and activate the known APRIL receptors BCMA and TACI. Collectively, the binding and signaling studies show that APRIL fused to Env retained its functionality and interacted with its known receptors BCMA and TACI efficiently. Binding of the APRIL domain of Env_APRIL_ to both receptors is important, because these receptors are thought to have distinct functions that are relevant for HIV-1 vaccine development. For example, signaling through TACI contributes to CSR [36], [37], whereas BCMA signaling is essential for long-lived bone marrow PC survival [28].

Rabbit immunizations proved that the APRIL domain was functional *in vivo* and enhanced the anti-Env Ab responses. We used rabbits for immunizations because rabbits, in contrast to mice, can produce Abs with long CDRH3 domains that are often found in HIV-1 bNAbs (reviewed in [1]). Furthermore, APRIL does not function very efficiently as an adjuvant in mice [18], [57], [58]. Env_APRIL_–immunized rabbits had significant increases in anti-gp120 binding Ab titers compared to Env_wt_ at week 6 (after the first boost), week 8, week 10 (after the second boost) and week 12 (terminal bleed). Our larger study here, using 12 animals per group, thus confirmed previous experiments using only 4 rabbits/group [13], and provided enough statistical power to confidently conclude that targeting Env to B cells using APRIL provides an advantage that should be further explored in HIV vaccine development.

In our previous studies in which we compared the immunogenicity of Env fused to the TNF superfamily members, APRIL, BAFF and CD40L, anti-APRIL/BAFF/CD40L responses were not investigated. We were triggered to study this when we found that Env fused to IL-21 or GM-CSF induced massive Ab responses against the self-molecules in both mice and rabbits [20]. Induction of Abs targeting and potentially neutralizing self-molecules might be perceived as a concern, although the reported effects of anti-cytokine responses in healthy individuals and individuals treated with cytokines therapeutically, have been both positive and negative [59]–[66]. Although several cytokines have been fused to HIV-1 Env molecules, only a few studies reported on the detection of Abs targeting the cytokine domain of the fusion proteins [19], [20], while other studies did not [11], [14]–[17]. Here, we found that Env_APRIL_ did not induce autoAbs targeting the APRIL domain in mice or rabbits and similar results were obtained with Env_CD40L_. These results were in stark contrast with the high anti-rGM-CSF and anti-rIL-21 responses observed in rabbits immunized with Env_IL-21_ and Env_GM-CSF_ [20]. The anti-APRIL responses were>250-fold lower than those against GM-CSF and>25-fold lower than those against IL21, suggesting that the type of cytokine (trimeric TNF superfamily member *vs.* monomeric 4-helix bundle cytokine) influences the propensity to generate autoAbs.

HIV-1 Env's intrinsic properties to limit the induction of bNAbs are multifold. Instability and conformational heterogeneity have for a long time prevented the generation of stable trimeric mimics of the native Env spike. As a result, most recombinant Env proteins used for vaccination purposes have been unstable and/or uncleaved between the gp120 and gp41 subunits, exhibiting a non-native conformation and exposing many non-neutralizing Ab (non-NAb) epitopes which might function as immune decoy [46], [67]–[69]. Recently, a stable mimic of the native Env spike, BG505 SOSIP.664, has been described [42]–[44], [70]. This third generation SOSIP protein, based on the clade A BG505 sequences, exposes most known epitopes for bNAbs while occluding those for non-NAbs. Although, the Env used in this study includes the SOSIP modifications (it was based on the first generation JR-FL SOSIP.R6 gp140 [41]), the addition of the trimerization domain and APRIL moiety renders the protein uncleaved and therefore it cannot adopt a native-like conformation [12], [13]. However, we recently found that APRIL can be added to the C-terminus of BG505 SOSIP.664 gp140 without adversely affecting its conformation. The combination of the native-like conformation of BG505 SOSIP.664 with the beneficial effects of APRIL provides further opportunity to improve the Ab response against HIV-1 Env. In particular, the potential of APRIL to enhance SHM, CSR, and PC survival is appealing.

## Conclusions

Here, we have further studied the function and immunogenicity of chimeric Env-APRIL fusion proteins. APRIL was used as a co-stimulatory molecule to improve the immunogenicity of Env. Env-APRIL molecule bound to and signaled through its known receptors BCMA and TACI that play important roles in humoral immunity. In rabbits, Env-APRIL improved the antibody responses against the Env compared to the unmodified Env, whereas negligible levels of anti-APRIL antibodies were induced. This study confirms and extends previous work and shows that fusion of Env-based immunogens to APRIL can improve Env immunogenicity and might help in designing HIV vaccines that induce protective humoral immunity.

## Supporting Information

Figure S1 **The Env_wt_, Env_hAPRIL_ and Env_rAPRIL_ proteins, transiently expressed in 293T cells, were immunoprecipitated with VRC01, PGT121, PGT126 and PGT135 and analyzed by reducing SDS-PAGE followed western blot using MAb PA1.** The migration of marker proteins is indicated.
(TIFF)

Figure S2 **Microscopic images of (A) BCMA:Fas and (B) TACI:Fas reporter cells after incubation with Env_hAPRIL_, Env_rAPRIL_ or controls for 12 h.** The ligand binding is directly associated with cell death. The supernatants used for TACI-Fas killing were concentrated eight times. Each condition was tested in duplicate and the images are representative for three independent experiments using proteins derived from three independent transfections.
(TIFF)
