# Supplementary figures and images for: Enhanced Immunogenicity of HIV-1 Envelope gp140 Proteins Fused to APRIL
Source: PLoS One. 2014 Sep 23;9(9):e107683. doi: 10.1371/journal.pone.0107683 (PMC4172553; doi:10.1371/journal.pone.0107683)

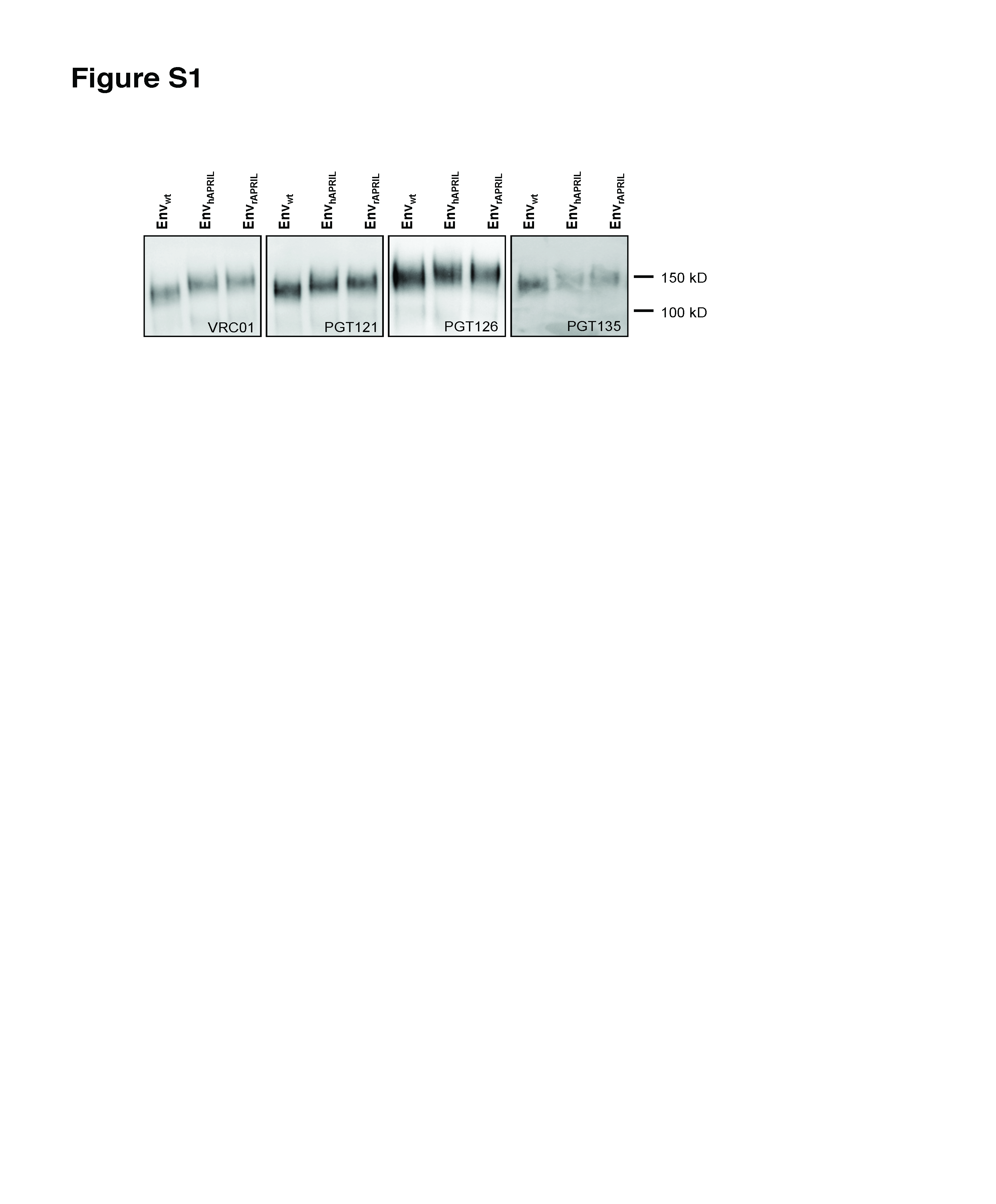

Supplement: Figure S1 — The Envwt, EnvhAPRIL and EnvrAPRIL proteins, transiently expressed in 293T cells, were immunoprecipitated with VRC01, PGT121, PGT126 and PGT135 and analyzed by reducing SDS-PAGE followed western blot using MAb PA1. The migration of marker proteins is indicated. (TIFF) [file pone.0107683.s001.tiff]

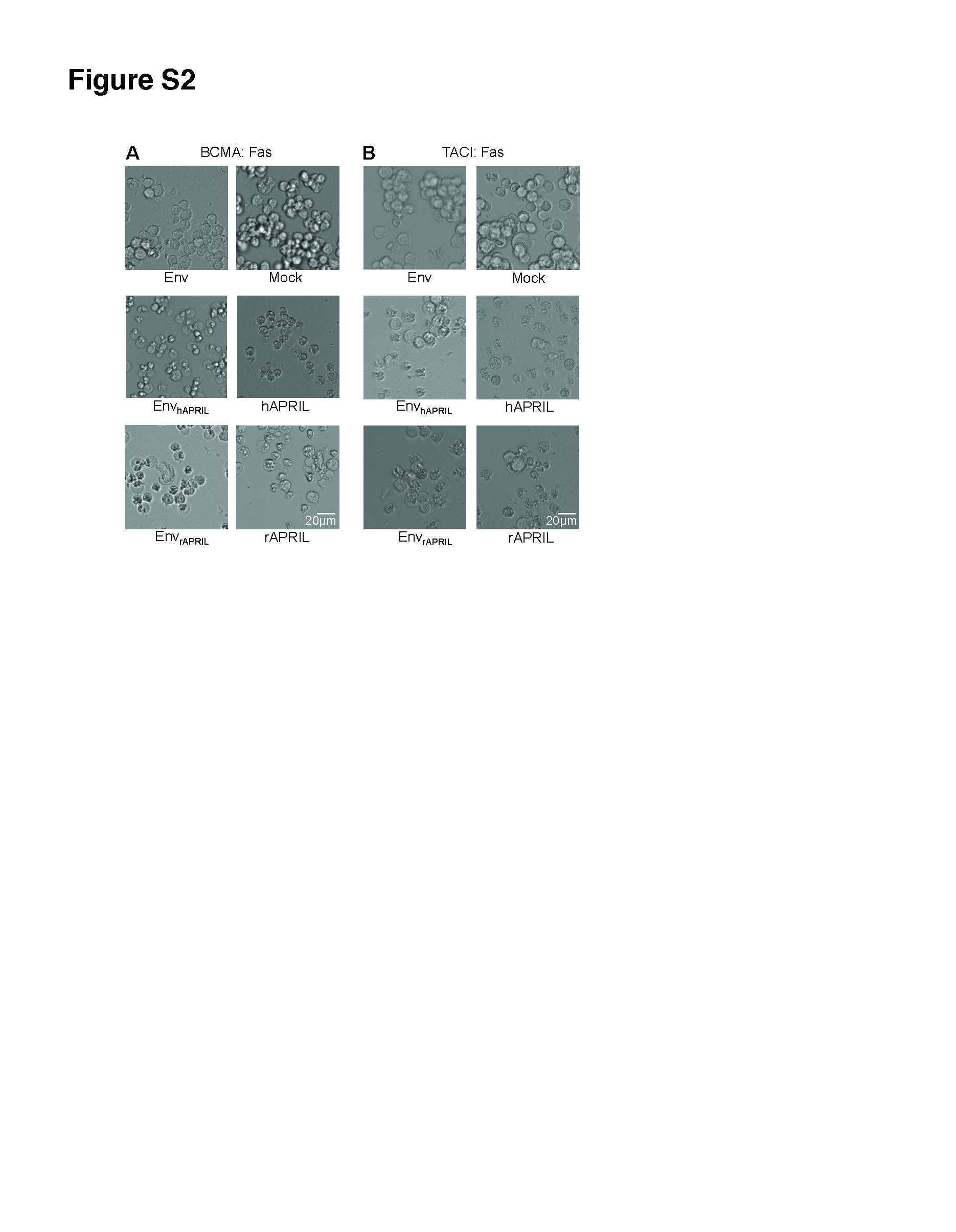

Supplement: Figure S2 — Microscopic images of (A) BCMA:Fas and (B) TACI:Fas reporter cells after incubation with EnvhAPRIL, EnvrAPRIL or controls for 12 h. The ligand binding is directly associated with cell death. The supernatants used for TACI-Fas killing were concentrated eight times. Each condition was tested in duplicate and the images are representative for three independent experiments using proteins derived from three independent transfections. (TIFF) [file pone.0107683.s002.tiff]
